# Complete Mitochondrial Genome of *Acheilognathus mengyangensis* (Cypriniformes, Cyprinidae, and Acheilognathinae): Characterization and Phylogenetic Analysis

**DOI:** 10.1002/ece3.71909

**Published:** 2025-08-03

**Authors:** Zhengran Li, Jinhui Yu, Chuanjiang Zhou

**Affiliations:** ^1^ The Observation and Research Field Station of Taihang Mountain Forest Ecosystems of Henan Province College of Life Sciences, Henan Normal University Xinxiang People's Republic of China

**Keywords:** *Acheilognathus mengyangensis*, mitochondrial genome, molecular evolution, phylogenetic relationships

## Abstract

In this study, the mitochondrial genome data of *Acheilognathus mengyangensis* were subjected to a multigene tandem method to elucidate its taxonomic status. The total length of the mitochondrial genome was determined to be 16,779 bp, including 13 protein‐coding genes, 2 rRNA genes, 22 tRNA genes, and the D‐loop region. Overall, there was a strong AT bias and anti‐G bias; different protein‐coding genes exhibited different degrees of codon preference. The rest of the amino acids, with the exception of tRNA^Ser (GCT)^, of the 22 tRNAs were in one form, the secondary structure was incomplete, and the remaining tRNAs folded to form the secondary structure of a typical clover. Thirteen protein‐coding genes of *Acheilognathus* and *Rhodeus* were concatenated to explore their phylogenetic relationships, with *Pseudorasbora parva* as the outgroup. The results revealed that *A. mengyangensis* , *Acheilognathus chankaensis* , and *Acheilognathus macropterus* were the most closely related. This research would supplement the *Acheilognathus* mitochondrial genome data, serving as a molecular basis for safeguarding species, facilitating genetic classification, and investigating Acheilognathinae phylogeny.

## Graphical abstract

This study presents the first complete mitochondrial genome of Acheilognathus mengyangensis, a freshwater fish species endemic to specific regions in China. Our work elucidates its genomic structure, codon usage patterns, and phylogenetic relationships within the Acheilognathinae subfamily. The findings provide critical molecular insights into species conservation, genetic classification, and evolutionary studies, addressing gaps in the current understanding of this understudied group. By comparing *A. mengyangensis* with closely related species, we highlight its taxonomic position and evolutionary constraints, contributing to broader discussions on mitochondrial genome evolution in teleost fishes.

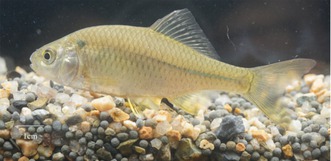

## 1 Introduction

Members of the Acheilognathinae subfamily primarily inhabit freshwater environments, such as rivers and streams, with a preference for areas with slower currents and clearer water. This subfamily includes 72 species (Tang et al. 2024), and the following 6 recognized genera: *Rhodeus* Agassiz (1832); *Tanakia* Jordan et al. (1914); *Paratanakia* Chang et al. (2014); *Acheilognathus* Bleeker (1860); *Pseudorhodeus* Chang et al. (2014); and *Sinorhodeus* Li et al. (2017). *Acheilognathus* is distributed broadly throughout China's primary freshwater networks, including the Yangtze, Pearl, Amur, and Yellow River basins. In this species, during reproduction, mature females form a tubular egg‐laying organ to transfer their ova into the gill cavities of freshwater mussels (*Unio douglasiae Griffith et Pidgeon*). Then, sperm from the male fish enter *Unio douglasiae Griffith et Pidgeon*'s inhalant siphon and gills to facilitate fertilization. The fertilized eggs subsequently hatch and develop within the mussel gills until the juveniles can swim independently (Smith et al. 2000; Ferraris Jr. 2003; Reichard et al. 2010; Yi et al. 2024).

Advances in genomic technologies have facilitated the characterization of diverse mitochondrial genomes (mtDNAs), providing critical insights into fish evolutionary history, population dynamics, and conservation prioritization (Moritz et al. 1987; Guo et al. 2004; Chen 2011). The mitochondrial genome of *A. mengyangensis* has been fully annotated to characterize its length, non‐coding regions, PCGs (protein‐coding genes), and RNA elements, with subsequent comparative analysis.

*A. mengyangensis* is a species of small fish typically found in slow‐moving streams and rivers (Figure S2). *A. mengyangensis* is generally about 5–8 cm in body length, and the body is flattened on the side and spindle‐shaped. The body color is usually silvery white or pale yellow; the dorsal and caudal fins are more developed, while the caudal fin is forked. *A. mengyangensis* mainly feeds on aquatic insects, algae, plankton, etc. The female fish of this species deposits eggs in the gills of *Unio douglasiae Griffith et Pidgeon*. To date, specimens of this species have been collected only from the Mengyang River in Mengyang town, Pengzhou city, and the tributaries of the Baitiao River in Tianma town, Dujiangyan city, both of which are located in Chengdu, Sichuan Province, China. In this study, the complete mtDNA sequences for bitterling fishes (*Acheilognathus* and *Rhodeus* genera) were retrieved from the NCBI database. *Pseudorasbora parva* was used as the outgroup for phylogenetic analysis. In total, 13 PCGs were concatenated, and ML and Bayesian phylogenetic trees were constructed using the best‐fit substitution model for nucleotide sequences and the optimal partitioning strategy, respectively. *A. mengyangensis* is a recently described species for which no mitogenomic data were previously available. Sequencing its mitochondrial genome provides foundational data to clarify its phylogenetic position within Acheilognathus, particularly given the taxonomic uncertainties surrounding this genus. Additionally, as an ornamental species with limited distribution and small population size, the characterization of its mitogenome serves as a critical reference for future conservation efforts, as accurate species delimitation is essential for effective management strategies.

## 2 Materials and Methods

### 2.1 Specimen Acquisition and Primary Data Production

*A. mengyangensis* samples were obtained from Mengyang Town, Chengdu City, Sichuan Province, China. No ethical approval or collection permits were required for the samples used in this study, as they were obtained from publicly accessible sources with no protected status under local regulations. The collected samples were fixed in 100% ethanol and archived in the Life Sciences Department of Henan Normal University. The georeferencing data were unavailable for this species due to incomplete site documentation during sampling. The genomic DNA of *A. mengyangensis* was isolated using standard phenol‐chloroform extraction protocols (Sambrook and Russell 2001). Muscle tissue was dissolved with proteinase K to release DNA, followed by the addition of a phenol‐chloroform mixture. After centrifugation, the aqueous layer was collected, and the DNA was precipitated with isopropanol or ethanol. The DNA pellet was then washed with ethanol and dried. The DNA samples were eluted in 40 μL of sterile distilled water and cryopreserved at −20°C. The complete mitochondrial genome of *A. mengyangensis* was obtained through high‐throughput coverage resequencing conducted by Personalbio (Nanjing, China) using the Illumina MiSeq platform. Raw reads were quality‐filtered and assembled to generate the final mitochondrial genome sequence.

Whole mitogenome alignment of *A. mengyangensis* and *A. tonkinensis* was performed, and MitoZ 3.4 (Meng et al. 2019) was applied for mitogenome assembly (GenBank file) and gene boundary annotation.

### 2.2 Annotation of the Mitochondrial Genome

Mitogenome reconstruction was performed using reference‐guided assembly against the existing Acheilognathinae sequences in NCBI. The start codon of the transfer RNA (tRNA^Phe^) was identified using the MITOS website (Bernt et al. 2013). Codon‐based preprocessing of the mitochondrial genes was conducted following the standard teleost genetic code, and the circular nature of the mitochondrial genome was verified. The Mitofish platform was utilized to determine the locations of 13 PCGs, 22 transfer RNAs (tRNAs), 2 ribosomal RNAs (rRNAs), and control regions, which generated a gene map for the *A. mengyangensis* mitochondrial genome. In order to annotate the tRNA genes, the tRNAscan‐SE algorithm (Lowe and Chan 2016) was applied, and the gene boundaries and generated cloverleaf secondary structure models were validated. For PCG translation, nucleotide sequences were aligned against the vertebrate mitochondrial genetic code in MEGA7.0 (Kumar et al. 2016), and manual verification of start/stop codons was performed. The base composition and relative synonymous codon usage (RSCU) were calculated using MEGA7.0. The AT:GC skew was calculated using Perna's formula (Perna and Kocher 1995), where AT Skew = (A − T)/(A + T) and GC Skew = (G − C)/(G + C). CodonW 1.4.2 software (Peden 2000) was employed to derive the codon adaptation index (Jin et al. 2024), effective number of codons (ENC), and GC and GC3 contents.

### 2.3 Phylogenetic Assessment

In order to investigate the phylogenetic position of *A. mengyangensis* , the dataset comprised 23 mitogenomes (18 *Acheilognathus* and 5 *Rhodeus*) curated from NCBI on the basis of assembly completeness (> 95% coverage). Taxon inclusion was performed following the most recent molecular phylogeny, confirming Gobioninae–Acheilognathae sisterhood, thereby minimizing the outgroup selection artifacts (Tang et al. 2024). *Pseudorasbora parva* (GenBank accession: NC_028016) was used as the outgroup, given its well‐established phylogenetic position as a sister taxon to Acheilognathinae within Cyprinoidea and its optimal genetic distance for reliable tree calibration. This gobionid species is widely used in prior studies, ensuring analytical consistency, while its extensive genomic data availability minimizes alignment artifacts. The final dataset included 23 mitogenomes, comprising 18 ingroup and 5 outgroup species, to ensure topological stability, and their GenBank accession numbers and corresponding species names are provided in Table S1. Two partitioned phylogenetic analysis methods were employed: one based on the Bayes principle (Ronquist and Huelsenbeck 2003) and the other on the maximum likelihood (ML) partitioned analysis method (Stamatakis 2006). Using PhyloSuit software PEVuZE5vdGU (Zhang et al. 2020), 13 PCGs, 2 ribosomal RNA (rRNA) genes, and 22 transfer RNA (tRNA) genes from each species were extracted. The extracted PCGs were then aligned individually using ClustalW (Peden 2000).

The sequences were aligned based on the mitochondrial genome using Phylosuite 1.2.3 (Zhang et al. 2020) to concatenate the PCGs into a 13‐PCG dataset. The optimal partitioning scheme and the corresponding nucleotide substitution model were determined using ModelFinder (Kalyaanamoorthy et al. 2017) based on the Akaike information criterion (AIC). A maximum likelihood (ML) tree was constructed using IQ‐TREE with an edge‐linked partitioning model and 5000 ultrafast bootstrap replicates (Stamatakis 2006; Lanfear et al. 2016). For Bayesian inference analyses, PartitionFinder (Lanfear et al. 2016) was employed to select the best partition and the associated optimal nucleotide substitution model according to the Bayesian information criterion (BIC) (Huelsenbeck and Ronquist 2001; Drummond and Rambaut 2007). Bayesian phylogenetic trees were inferred using MrBayes 3.2.6 (Ronquist and Huelsenbeck 2003) under the N/A model, with two parallel runs of 2,000,000 iterations. The initial 25% of the sampled trees were discarded as burn‐in.

## 3 Results

### 3.1 Genome Size and Organization

Assembly of the *A. mengyangensis* mitogenome yielded a circular molecule 16,779 bp in length (Figure S3) and exhibiting a conserved vertebrate gene repertoire: 13 PCGs (ranging from 165 bp for ATP8 to 1836 bp for ND5), 2 rRNAs, and 22 tRNAs. Notably, COX1 (1581 bp) and CYTB (1141 bp) presented the highest sequence conservation (> 90% identity) with other Cyprinidae species, 2 ribosomal RNA (rRNA) genes, and 22 transfer RNA (tRNA) genes, totaling 37 genes. The gene distribution analysis revealed pronounced strand asymmetry: while 28 genes (including 12 PCGs and 16 RNAs) were encoded on the heavy strand (H‐strand), only a minor cluster of 8 tRNAs and ND6 (522 bp) was located on the light strand (L‐strand). This gene arrangement was consistent with the typical genetic organization observed in teleost fish (Figure S3, Table S2).

### 3.2 Base Composition

In *A. mengyangensis* , the highest base content of 29.52% was observed for A, followed by T (27.21%), C (26.28%), and G (16.99%). Strong AT preference (56.7%) and guanine depletion (16.9%) were observed (Table S3), reflecting the selection of energy‐favorable base pairs in the mitochondrial transcripts. This compositional bias was similar to that observed in most sequenced hard bone mitotic genomes (Perna and Kocher 1995). Mitogenome‐wide nucleotide analysis revealed consistent AT richness (D‐loop: 72.1%, tRNAs: 61.3%, PCGs: 56.7%) with strand‐specific biases: 12/13 PCGs presented anti‐A bias (except ATP8) and 11/13 anti‐G bias (except ND2/ND3), whereas rRNAs presented extreme A bias (AT skew > 0.25) in contrast to the unique G bias of tRNAs. Notably, ND6 shared tRNA‐like G/T‐bias patterns, and PCGs demonstrated stronger anti‐G bias than D‐loops or rRNAs, suggesting that transcriptional strand asymmetry governed the regional composition differences.

### 3.3 Protein‐Coding Genes

The 13 protein‐coding genes in the *A. mengyangensis* mitogenome spanned 11,421 bp, accounting for more than two‐thirds (68.07%) of its total genomic content.

#### 3.3.1 Use of Amino Acids and Codons

The 13 PCGs encoded 3798 amino acids, among which Leu (18.2%), Ser (12.1%), Pro (10.7%), and Thr (9.8%) dominated the composition (Table S4). Codon usage analysis (Figure S4) revealed a strong preference for AT: high‐frequency codons (AAA‐Lys, AAU‐Asn, and UAA‐Stop) contained 65%–72% A/U, whereas C/G‐rich codons (GGG‐Gly, GGC‐Gly, and CGC‐Arg) accounted for < 5% of the occurrences. The RSCU comparisons revealed that the most frequently used codons in *A. mengyangensis* were GCC (1.74%), UAA (1.56%), CAA (1.38%), and UUA (1.36%), which encoded the amino acids Ala, Tyr, Glu, and Leu, respectively. Conversely, the least frequently used codons encoded alanine (GCG, 0.33%), serine (UCG, 0.45%), and threonine (ACG, 0.45%).

#### 3.3.2 Start Codon and Stop Codon

In *A. mengyangensis* , most PCGs initiated translation with ATG (start codon), with the COX1 gene being the only exception, which used GTG as the start codon. ATP8, COX1, ND6, and ND4L utilized TAA as the complete stop codon, while ND1 and ND5 employed TAG as the complete stop codon. In contrast, COX2, COX3, ND2, ND3, ND4, ATP6, and CYTB used incomplete stop codons, specifically TA‐ and T‐‐.

#### 3.3.3 Synonymous Codon Preference

Synonymous codon preference (SCP) arises from the differential selection of the translationally equivalent codons, which is driven by mutational drift and functional optimization pressures. Generally, higher gene expression is associated with stronger SCP. In order to explore the codon usage preferences in the mitochondrial genome of *A. mengyangensis* , the codon adaptation index (Jin et al. 2024), effective number of codons (ENC), GC content, and GC content at the third codon position (GC3) were calculated using CodonW 1.4.2 software. These metrics provided insights into the evolutionary and functional constraints shaping codon usage preferences in this species.

The effective number of codons (ENC) reflects the extent to which codon usage deviates from random selection, and values typically range from 20 to 61. In the mitochondrial genome of *A. mengyangensis* , the ENC values for PCGs were observed to be in the range of 36.93 to 57.39 (Table S5), with most values falling within the 45–50 range. These findings indicated a moderate degree of codon usage preference in the mitochondrial genome of *A. mengyangensis* . The codon adaptation index (Jin et al. 2024) values range from 0 to 1, with high values indicating high gene expression levels and pronounced SCP (Sharp and Li 1987). Among the PCGs in *A. mengyangensis* , distinct codon usage patterns were observed among the PCGs: while ATP8 and cytochrome oxidase subunits (COX1–3) presented increased synonymous codon preference (SCP > 1.8) and high expression, ATP6 presented minimal bias (SCP < 0.5) and the lowest transcriptional activity (Table S5). Examination of third‐position GC content (GC3) across all codons, excluding those encoding methionine, tryptophan, and stop signals, demonstrated consistently decreased GC3 levels (mean 22.4% ±3.1%) in all 13 PCGs, with the G/C nucleotides occurring 2.3‐fold less frequently than expected at the wobble positions (*p* < 0.01). These findings further support the strong preference for AT observed in the *A. mengyangensis* PCGs (Table S3).

### 3.4 tRNA and rRNA

The mitochondrial genome encoded two ribosomal RNA components: one was a highly conserved 16S rRNA (large subunit) and the other was a more variable 12S rRNA (small subunit). The structural conservation between the two components exceeds their sequence similarity. The 958 bp 12S rRNA locus, positioned between tRNA^Phe^ and tRNA^Val^, constituted 5.71% of the mitogenome and displayed a balanced nucleotide composition (AT = 50.57%).

*A. mengyangensis* contained 22 tRNA genes (8 on L‐strand, 14 on H‐strand), ranging from 69 to 77 bp. Most formed canonical cloverleaf structures, while tRNASer (GCT) lacked the D‐arm (Figure S1)—a feature seen in other fish mitogenomes (Broughton et al. 2001; Hwang et al. 2013). Such atypical tRNAs may rely on co‐evolutionary adaptations or RNA editing for functionality. These mechanisms ensure appropriate tRNA functioning despite structural deviations (Masta and Boore 2004).

### 3.5 Comparative Ka/Ks Analysis of *A. mengyangensis*

The evolutionary selection pressures on mitochondrial protein‐coding genes were assessed through pairwise Ka/Ks (*ω*) analyses using KaKs Calculator 3.0 (Zhang 2021) between *A. mengyangensis* and seven representative species, including its closest relatives *A. chankaensis* , *A. macropterus* , and *A. yamatsutae* , the comparative taxa *R. notatus* , *T. lanceolata* , and *T. limbata* , and the outgroup ( *P. parva* ). Statistical comparisons based on one‐way ANOVA with Bonferroni‐corrected post hoc tests were conducted (*α* = 0.05, correction threshold = 0.0024 for 21 comparisons).

The results revealed a dominant pattern of purifying selection across all the mitochondrial genes (*ω* < 1, mean ± SD = 0.12 ± 0.04, *n* = 13), indicating strong functional constraints. Notably, the highly conserved cox1 gene presented the lowest *ω* value (0.004 ± 0.0001), which is consistent with its critical role in oxidative phosphorylation. One‐way ANOVA confirmed significant overall variation in the *ω* ratios across lineages (*F*_₆,₈₄_ = 8.74, *p* < 0.0001). The *ω* values of the three *Acheilognathus* species were statistically indistinguishable (mean range: 0.023–0.030, all pairwise *p* > 0.42 after correction), reinforcing their shared evolutionary constraints. In contrast, *R. notatus* (*ω* = 0.15 ± 0.02) presented significantly greater values than each of the *Acheilognathus* species evaluated (Bonferroni‐corrected *p* < 0.002), whereas *P. parva* (*ω* = 0.21 ± 0.03) differed significantly from *A. chankaensis* (*p* = 0.001) and *A. macropterus* (*p* = 0.0003). This stepwise increase in the *ω* values with phylogenetic distance was consistent with the neutral theory prediction that functional constraints weaken in distant lineages. Among the 13 protein‐coding genes, nad2 presented the greatest intergroup divergence (*Acheilognathus*: 0.051–0.083 vs. *R. notatus* : 0.083 ± 0.005), potentially indicating lineage‐specific evolutionary dynamics, although all values remained below 1. Notably, two of the comparisons— *A. chankaensis* vs. *T. lanceolata* (*p* = 0.021) and *R. notatus* vs. *T. limbata* (*p* = 0.025)—showed marginal significance after strict correction, suggesting subtle variations in selective pressures among non‐congeneric taxa. However, the overall pattern confirmed purifying selection as the dominant force, with no evidence of positive selection at the whole‐gene level (all *ω* < < 1) (Figure S5). Non‐synonymous replacement rate (Ka), synonymous replacement rate (Ks), and Ka/Ks values of the PCGs of *A. mengyangensis* and other Acheilognathinae species.

These findings support the prevailing view that mitochondrial protein‐coding genes are under strong purifying selection owing to their essential metabolic functions. While lineage‐specific *ω* differences suggested varying constraint intensities, future studies should employ branch‐site models to investigate potential adaptive evolution at specific codons that could have been masked in the whole‐gene analyses conducted in this study.

### 3.6 Phylogeny and Systematics of *A. mengyangensis*

The phylogenetic relationships of *A. mengyangensis* were investigated through the construction of maximum likelihood (ML) and Bayesian (MrBayes) trees by concatenating 13 PCGs and using *Pseudorasbora parva* as the outgroup. The best partitioning models for the ML and Bayesian analyses were determined based on the Akaike information criterion (AIC) and Bayesian information criterion (BIC), respectively, using the most suitable nucleotide substitution models for 34 concatenated PCG sequences.

In the ML analysis, the optimal partitioning models used were as follows:

GTR + F + I + G4 for ND2, ND5, ND6, COXI and COXIII.

GTR + F + I + I + R3 for ND1, ND3, ND4, ND4L, ATP6, and COXII.

TNe + I + G4 for Cytb.

HKY + F + G4 for ATP8.

The optimal partitioning models for the Bayesian analysis were slightly different, leading to minor variations in the node support values. While the overall topological structure of the trees was consistent, some nodes presented differences in support rates or posterior probabilities. These discrepancies were attributed to the different algorithms used in the ML and Bayesian analyses, which could have influenced the resulting phylogenetic trees.

The above results supported the conclusion that *Acheilognathus* and *Rhodeus* form monophyletic groups (Qing 2010). Specifically, *A. mengyangensis* was revealed to be most closely related to *Acheilognathus chankaensis* and *Acheilognathus macropterus* . This conclusion was consistent with the results of the Ka/Ks analysis. Both ML and MrBayes trees presented high node support rates for these relationships (81/0.749) (Figures S6 and S7, respectively).

## 4 Discussion and Conclusions

The complete mitochondrial genome of *A. mengyangensis* was assembled and annotated in this study, revealing a 16,779 bp circular DNA molecule, which is about the length reported for other Acheilognathinae species, such as *A. chankaensis* (16,774 bp), *A. rhombeus* (16,780 bp), and *A. omeiensis* (16,774 bp). Divergence in terms of genome sizes likely reflected copy number variation in the repetitive elements located in regulatory regions (Wang et al. 2020). Consistent with the typical mitochondrial genome structure of other teleost fishes, the *A. mengyangensis* mitochondrial genome was revealed to comprise 13 PCGs, 2 ribosomal RNA (rRNA) genes, 22 transfer RNA (tRNA) genes, a non‐coding control region (D‐loop), and an origin of light‐strand replication (OL). The majority of these genes were located on the heavy strand (H‐strand), with only ND6 and 8 tRNAs located on the light strand (L‐strand). This gene arrangement was consistent with the typical genetic organization observed in the Acheilognathinae subfamily (Hwang et al. 2013, 2012; Zhu et al. 2021). The base composition analysis revealed significant nucleotide bias in the *A. mengyangensis* mitogenome: the AT content (56.7%) substantially exceeded the GC content (43.3%), and guanine representation was particularly low (16.9%) (Table S3). This pattern was consistent with the base composition observed in the mitochondrial genomes of most of the other teleost fishes (Perna and Kocher 1995).

An analysis of the synonymous codon usage frequency in the *A. mengyangensis* mitochondrial genome revealed that the RSCU values for UUU, AUU, and UUA were greater than 1 (Figure S4). These findings indicated a preference for these codons in the mitochondrial genome of *A. mengyangensis* .

In addition, the phylogenetic relationships of *A. mengyangensis* were reconstructed by concatenating 13 PCGs. The phylogenetic results revealed topological differences compared to those reported in previous studies, and these differences could be attributed to variations in the choice of outgroups, comparative species, molecular markers, and individual gene sequences. These factors highlighted the complexity of phylogenetic analysis and the need for comprehensive approaches to achieve more accurate and consistent results (Chang et al. 2014; Qing 2010; Cheng et al. 2014; Miyake et al. 2021). Owing to the limited research currently available on *A. mengyangensis* , it was challenging to conduct a further comprehensive comparative analysis of this species. The results of the reconstructed phylogenetic tree indicated that *A. mengyangensis* is most closely related to *Acheilognathus chankaensis* and *Acheilognathus macropterus* . The node support rates for this relationship, determined using maximum likelihood (ML) and MrBayes analyses, were 0.749 and 81, respectively (Figures S6 and S7).

Compared to previous studies, this research utilized PCGs from the mitochondrial genome to construct a phylogenetic tree, providing a further comprehensive set of gene loci and a gene tree that reflected the species tree more accurately. However, as technology continues to advance, discrepancies between gene trees and species trees are becoming increasingly apparent. These discrepancies may arise from factors such as incomplete lineage sorting, hybridization, and gene flow, and further complicate the interpretation of phylogenetic relationships (Zhang et al. 2024). Therefore, to establish precise and reliable phylogenetic relationships, incorporating supplementary data sources, such as next‐generation sequencing (NGS) and reduced‐representation genomic data, is essential. These advanced approaches would provide a broader and more detailed genetic perspective, helping address the discrepancies between gene trees and species trees and improving the accuracy of phylogenetic inference.

This study presents the complete mitochondrial genome sequence and the associated characteristics of *A. mengyangensis* . A detailed analysis of the gene structure, RNA secondary structure, D‐loop region, and base composition was conducted. The findings not only enrich the mitochondrial genome database for *Acheilognathus* but also provide valuable insights at molecular and genetic levels for species conservation, molecular identification, and evolutionary studies in the context of the Acheilognathinae subfamily.

## Author Contributions

**Zhengran Li:** data curation (lead), writing – original draft (lead). **Jinhui Yu:** conceptualization (supporting), data curation (supporting). **Chuanjiang Zhou:** conceptualization (lead), writing – review and editing (lead).

## Ethics Statement

All experimental procedures received prior approval from the Institutional Animal Ethics Committee of Henan Normal University and were conducted in strict accordance with: National Standard GB/T 35892‐2018 requirements (https://www.htu.edu.cn/xswyh/2025/0221/c12459a336306/page.htm). ARRIVE 2.0 reporting guidelines. International Council for Laboratory Animal Science (ICLAS) ethical benchmarks. Henan Normal University Scientific Research Ethics Review and Approval Form (No.: HNSD‐2024BS‐1013). Continuous monitoring ensured full compliance throughout the study duration.

## Conflicts of Interest

The authors declare no conflicts of interest.

## Supporting information


**Figure S1:** ece371909‐sup‐0001‐FigureS1.docx.

**Figure S2:** Image showing the morphological appearance of *A. mengyangensis* . The photo was captured by Jinhui Yu.

**Figure S3:** Gene map of the *A. mengyangensis* mitochondrial genome.

**Figure S4:** Analysis of RSCU in the mitochondrial genome of *A. mengyangensis* . “*” denotes stop codon.

**Figure S5:** Non‐synonymous replacement rate (Ka), synonymous replacement rate (Ks), and Ka/Ks values of the PCGs of *A. mengyangensis* and other Acheilognathinae species.

**Figure S6:** Phylogenetic tree constructed using the Bayesian approach.

**Figure S7:** Phylogenetic tree constructed using the maximum likelihood (ML) approach.

**Table S1:** Genome sequences from NCBI used in this study.

**Table S2:** Mitochondrial genes and the associated features of *A. mengyangensis* .

**Table S3:** Base composition analysis of the *A. mengyangensis* mitogenome regions.

**Table S4:** Analysis of RSCU for the PCGs of the mitochondrial genome of *A. mengyangensis* .
Note: “*” denotes a stop codon.

**Table S5:** Preference for codon usage in the protein‐encoding genes in the mitochondrial genome of *A. mengyangensis* .

## Data Availability

The original data presented in the study are openly available in CNSA at CNP0006862.
