# Supplementary material for: Complete Mitochondrial Genome of Acheilognathus mengyangensis (Cypriniformes, Cyprinidae, and Acheilognathinae): Characterization and Phylogenetic Analysis
Source: Ecol Evol. 2025 Aug 3;15(8):e71909. doi: 10.1002/ece3.71909 (PMC12318612; doi:10.1002/ece3.71909)
Supplement: Supplementary file 1 — Figure S1: ece371909‐sup‐0001‐FigureS1.docx. [file ECE3-15-e71909-s010.docx]

Supplementary Material

**
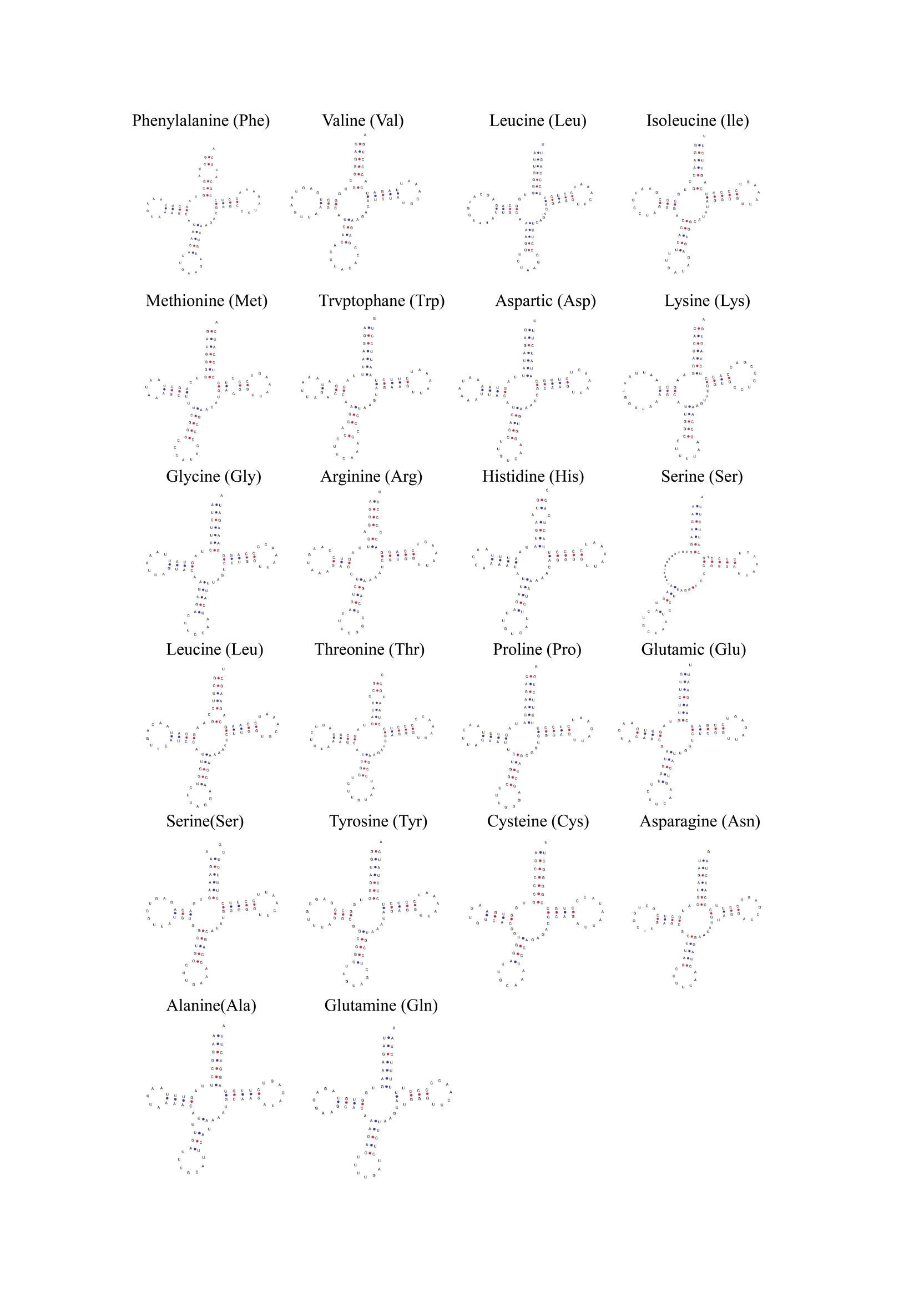
**

**Figure S1.** Secondary structure of the 22 tRNA genes in the mitochondrial genome of *A. mengyangensis.*
