# Supplementary material for: Complete Mitochondrial Genome of Acheilognathus mengyangensis (Cypriniformes, Cyprinidae, and Acheilognathinae): Characterization and Phylogenetic Analysis
Source: Ecol Evol. 2025 Aug 3;15(8):e71909. doi: 10.1002/ece3.71909 (PMC12318612; doi:10.1002/ece3.71909)
Supplement: Supplementary file 2 — Figure S2: Image showing the morphological appearance of A. mengyangensis . The photo was captured by Jinhui Yu. [file ECE3-15-e71909-s001.pdf]

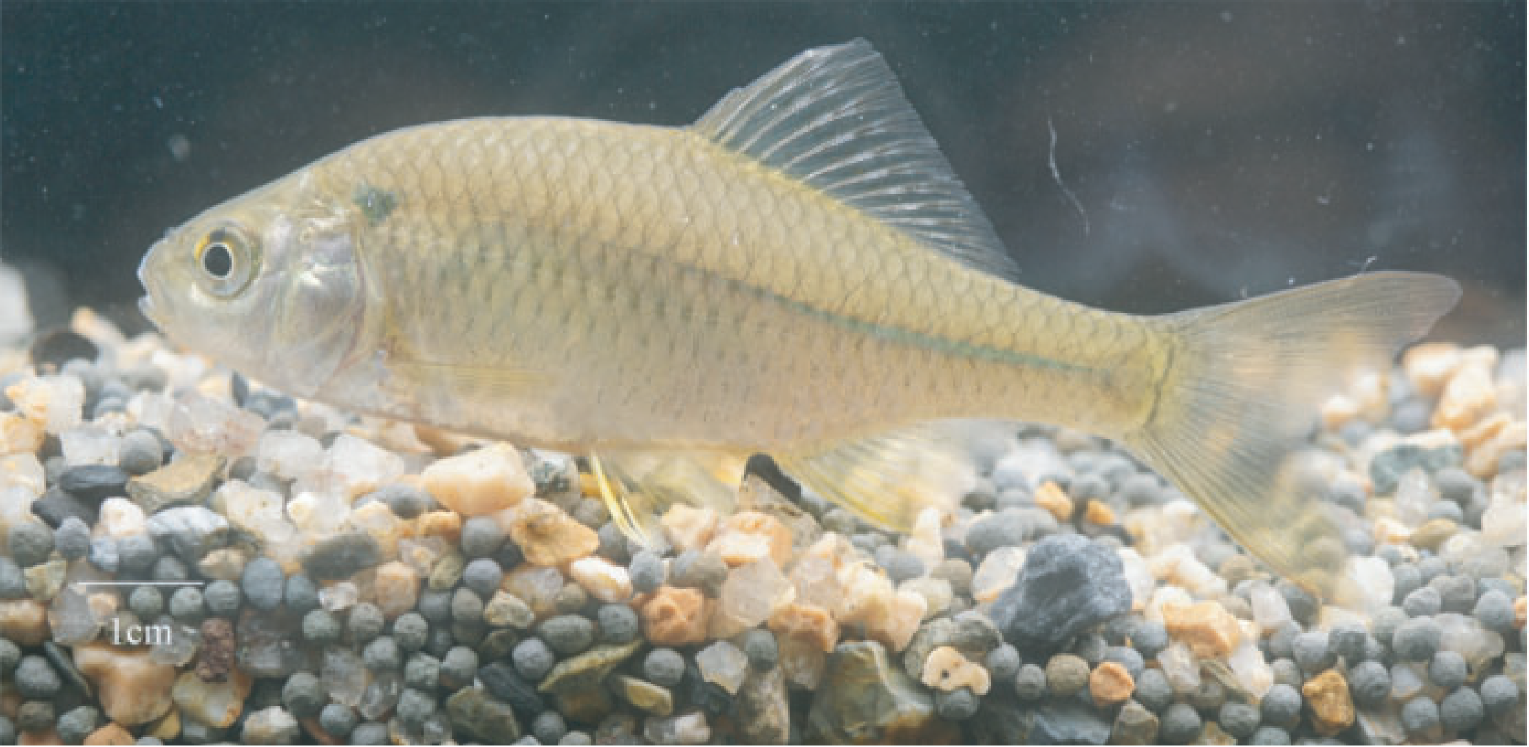

Figure S2. Image showing the morphological appearance of *A. mengyangensis*. The photo was captured by Jinhui Yu.
