# Supplementary figures and images for: Complete Mitochondrial Genome of Acheilognathus mengyangensis (Cypriniformes, Cyprinidae, and Acheilognathinae): Characterization and Phylogenetic Analysis
Source: Ecol Evol. 2025 Aug 3;15(8):e71909. doi: 10.1002/ece3.71909 (PMC12318612; doi:10.1002/ece3.71909)

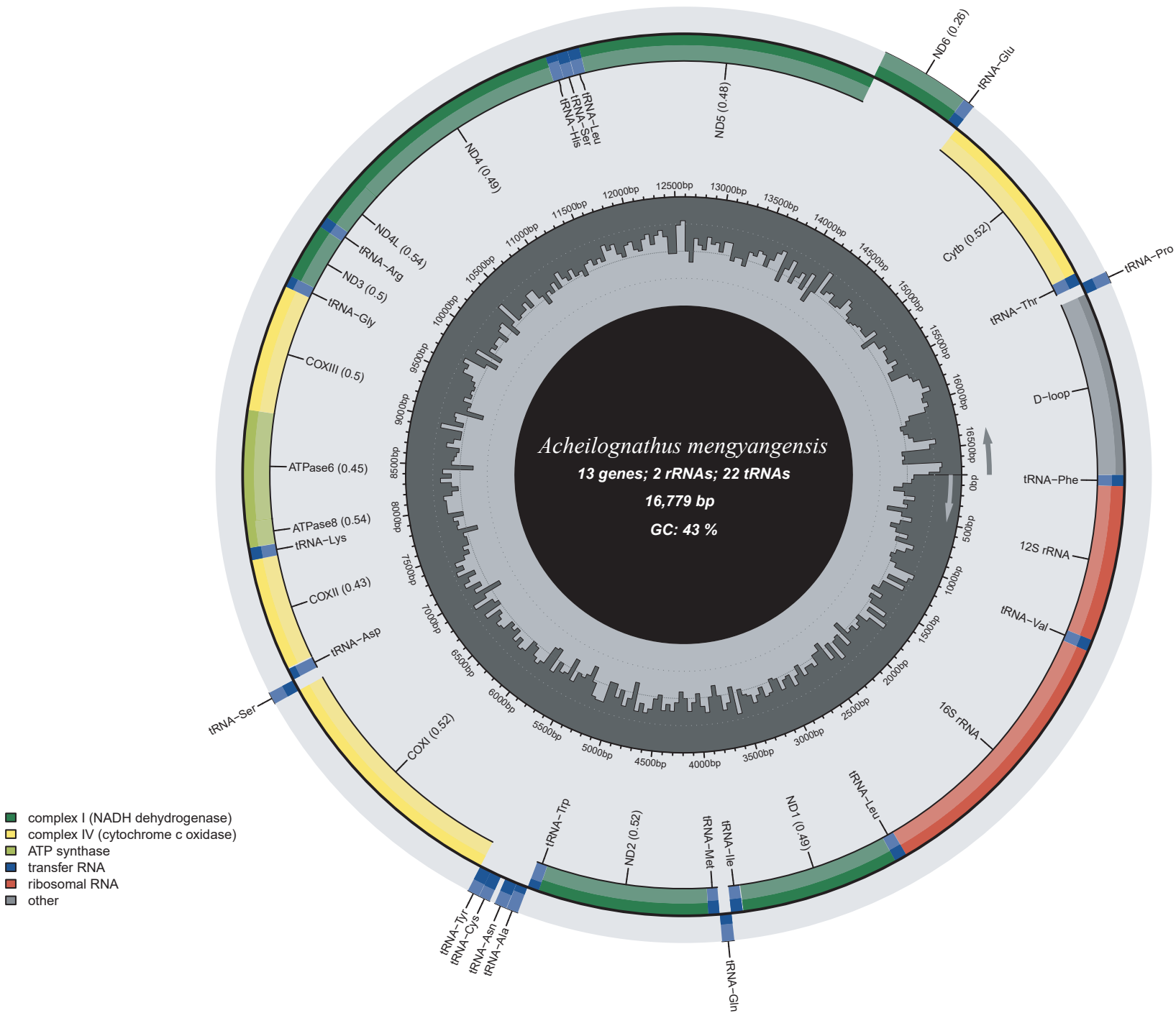

**Figure S3.** Gene map of the *A. mengyangensis* mitochondrial genome.

Supplement: Supplementary file 3 — Figure S3: Gene map of the A. mengyangensis mitochondrial genome. [file ECE3-15-e71909-s007.pdf]

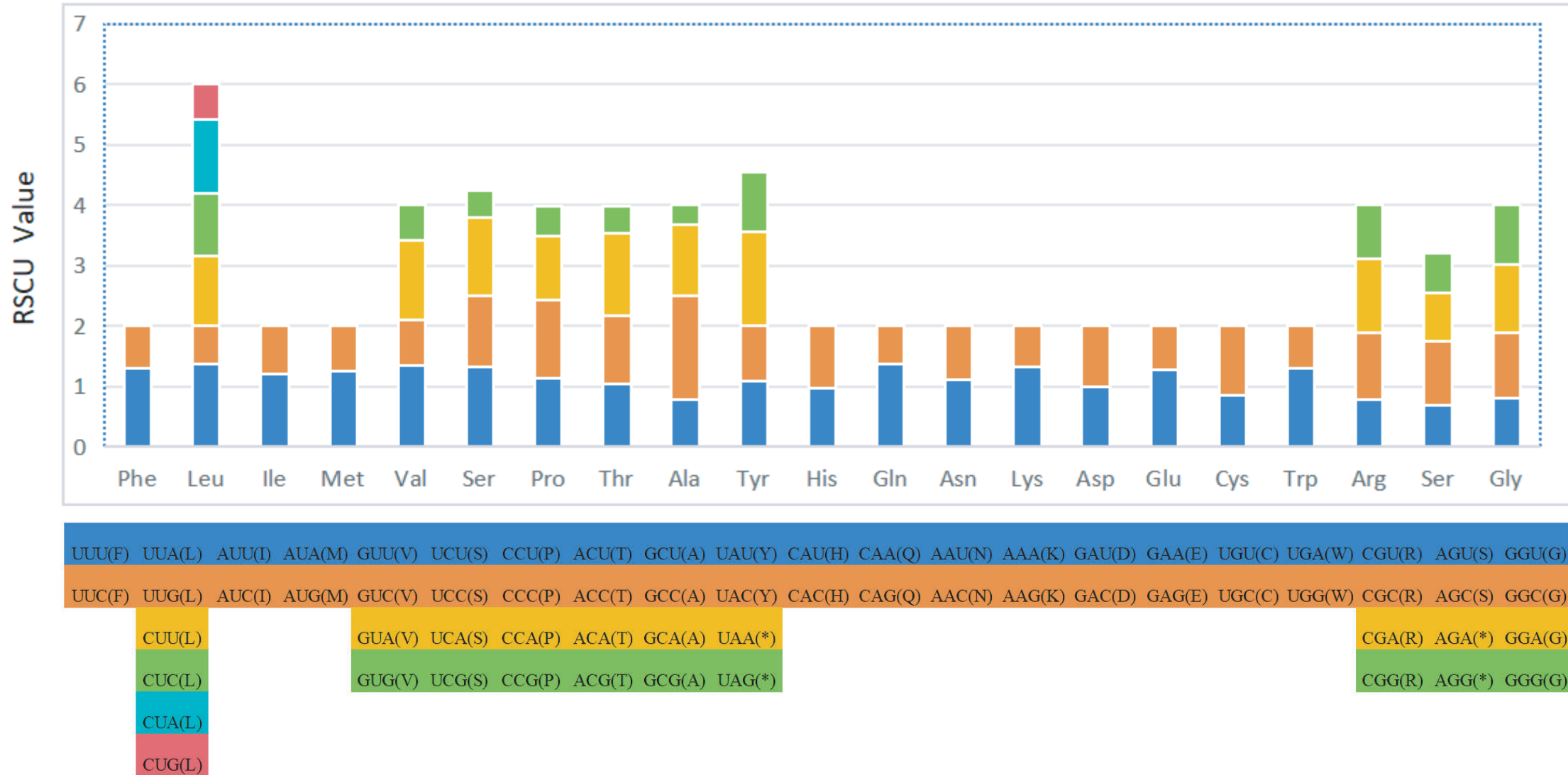

**Figure S4.** Analysis of RSCU in the mitochondrial genome of *A. mengyangensis*. “\*” denotes a stop codon.

Supplement: Supplementary file 4 — Figure S4: Analysis of RSCU in the mitochondrial genome of A. mengyangensis . “*” denotes stop codon. [file ECE3-15-e71909-s005.pdf]

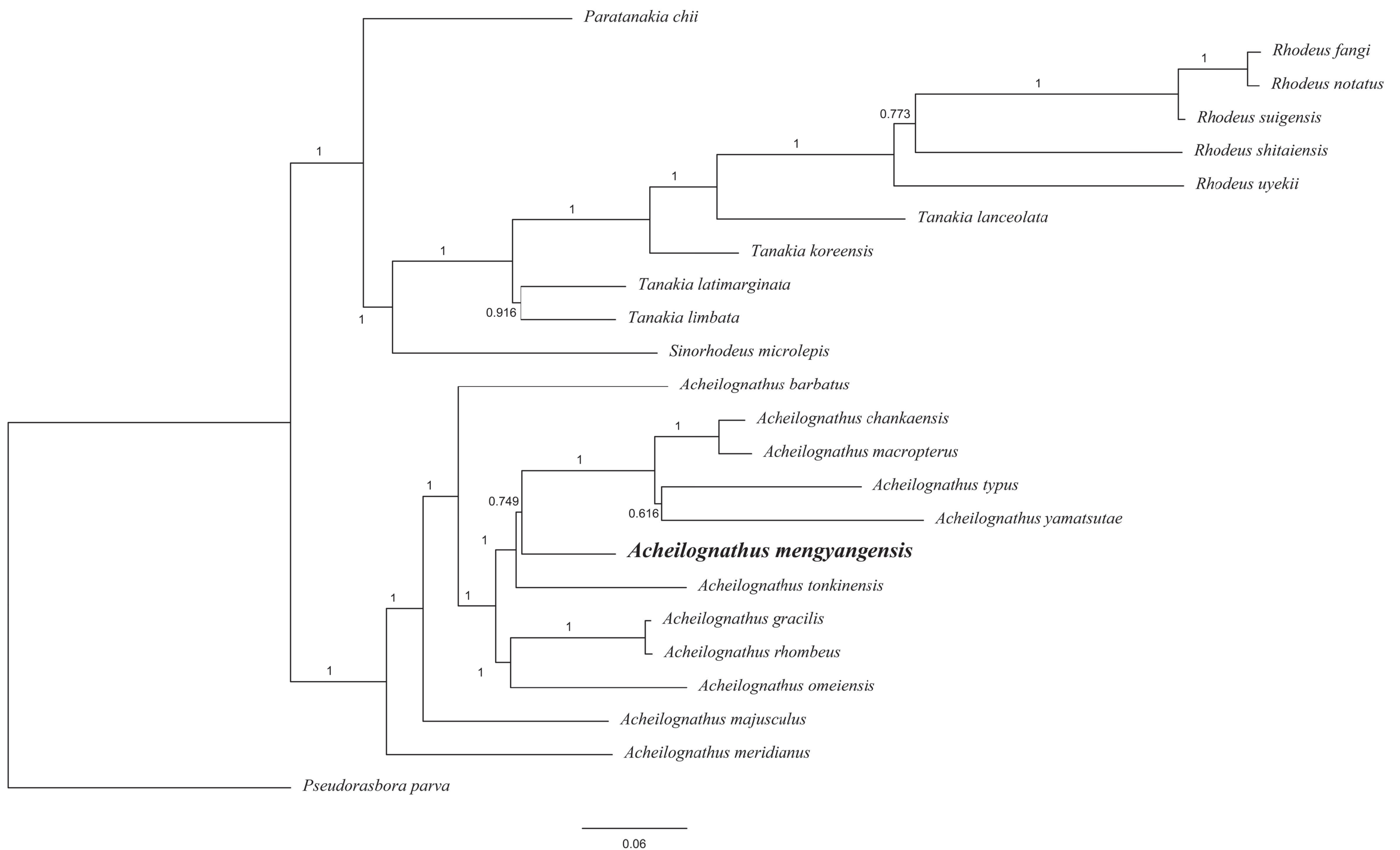

**Figure S6.** Phylogenetic tree constructed using the Bayesian approach.

Supplement: Supplementary file 6 — Figure S6: Phylogenetic tree constructed using the Bayesian approach. [file ECE3-15-e71909-s012.pdf]

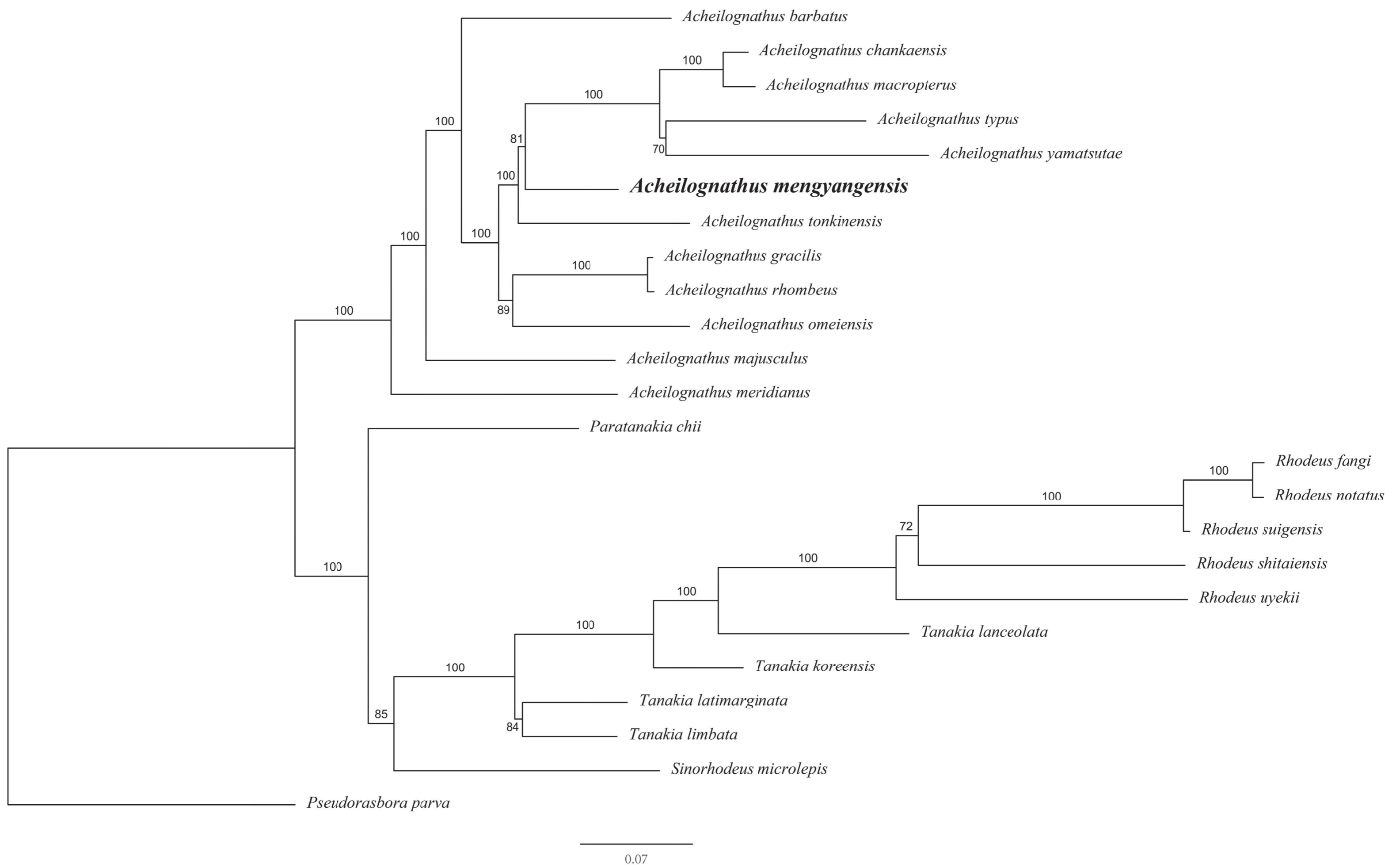

Supplement: Supplementary file 7 — Figure S7: Phylogenetic tree constructed using the maximum likelihood (ML) approach. [file ECE3-15-e71909-s006.pdf]
