# Supplementary material for: Complete Mitochondrial Genome of Acheilognathus mengyangensis (Cypriniformes, Cyprinidae, and Acheilognathinae): Characterization and Phylogenetic Analysis
Source: Ecol Evol. 2025 Aug 3;15(8):e71909. doi: 10.1002/ece3.71909 (PMC12318612; doi:10.1002/ece3.71909)
Supplement: Supplementary file 5 — Figure S5: Non‐synonymous replacement rate (Ka), synonymous replacement rate (Ks), and Ka/Ks values of the PCGs of A. mengyangensis and other Acheilognathinae species. [file ECE3-15-e71909-s011.pdf]

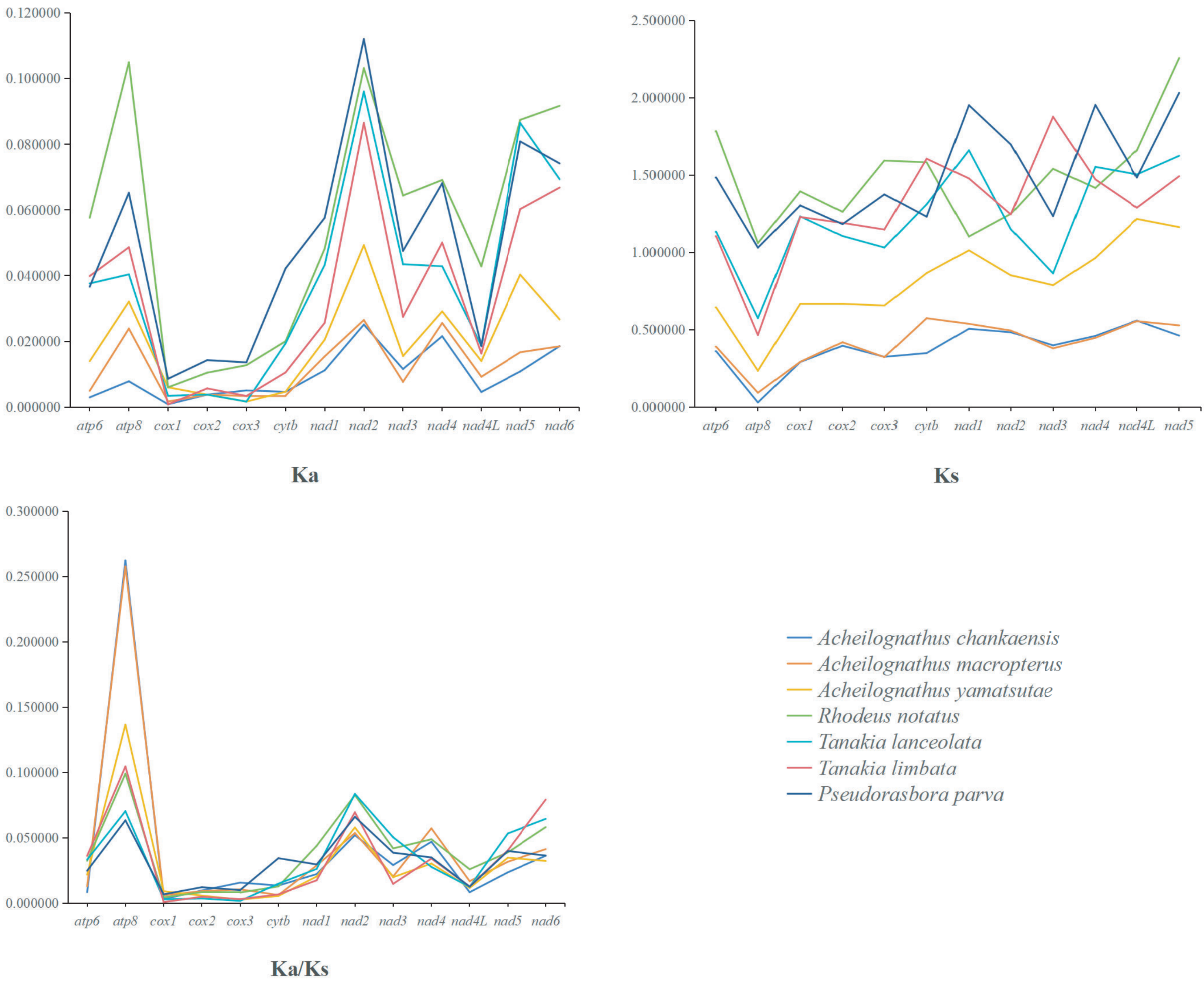

Figure S5. Non-synonymous replacement rate (Ka), synonymous replacement rate (Ks), and Ka/Ks values of the PCGs of *A. mengyangensis* and other Acheilognathinae species.
