# Supplementary material for: Complete Mitochondrial Genome of Acheilognathus mengyangensis (Cypriniformes, Cyprinidae, and Acheilognathinae): Characterization and Phylogenetic Analysis
Source: Ecol Evol. 2025 Aug 3;15(8):e71909. doi: 10.1002/ece3.71909 (PMC12318612; doi:10.1002/ece3.71909)
Supplement: Supplementary file 8 — Table S1: Genome sequences from NCBI used in this study. [file ECE3-15-e71909-s003.docx]

**Table S1.** Genome sequences from NCBI used in this study.

| GenBank Accession | Species | Length | AT (%) |
| --- | --- | --- | --- |
| NC007885 | *Rhodeus uyekii* | 16817 | 55 |
| NC008668 | *Acheilognathus typus* | 16778 | 57 |
| NC013704 | *Tanakia koreensis* | 16563 | 54 |
| NC013709 | *Rhodeus suigensis* | 16733 | 55.1 |
| NC013711 | *Acheilognathus macropterus* | 16774 | 57 |
| NC013712 | *Acheilognathus yamatsutae* | 16703 | 56.7 |
| NC022690 | *Rhodeus shitaiensis* | 16774 | 55 |
| NC023101 | *Acheilognathus chankaensis* | 16774 | 58 |
| NC024566 | *Tanakia lanceolata* | 16607 | 54 |
| NC025515 | *Tanakia limbata* | 16565 | 54 |
| NC026872 | *Acheilognathus barbatus* | 16770 | 56 |
| NC027437 | *Rhodeus fangi* | 16733 | 55.3 |
| NC028416 | *Acheilognathus gracilis* | 16988 | 57 |
| NC028433 | *Acheilognathus rhombeus* | 16780 | 56.9 |
| NC028736 | *Acheilognathus majusculus* | 17155 | 57 |
| NC029718 | *Rhodeus notatus* | 16735 | 55.3 |
| NC031152 | *Acheilognathus meridianus* | 16563 | 57.9 |
| NC037404 | *Acheilognathus omeiensis* | 16774 | 56.7 |
| NC039820 | *Tanakia latimarginata* | 16588 | 54 |
| NC042407 | *Acheilognathus tonkinensis* | 16767 | 56.5 |
| NC042717 | *Sinorhodeus microlepis* | 16591 | 57 |
| NC066655 | *Paratanakia chii* | 16575 | 57 |
| NC015614 | *Pseudorasbora parva* | 16600 | 58.9 |
