# Supplementary material for: Complete Mitochondrial Genome of Acheilognathus mengyangensis (Cypriniformes, Cyprinidae, and Acheilognathinae): Characterization and Phylogenetic Analysis
Source: Ecol Evol. 2025 Aug 3;15(8):e71909. doi: 10.1002/ece3.71909 (PMC12318612; doi:10.1002/ece3.71909)
Supplement: Supplementary file 9 — Table S2: Mitochondrial genes and the associated features of A. mengyangensis . [file ECE3-15-e71909-s004.docx]

**Table S2.** Mitochondrial genes and the associated features of *A. mengyangensis*.

|  | Type | One-letter code | Strand | Amino acids | Position | | | Codon | | |
| --- | --- | --- | --- | --- | --- | --- | --- | --- | --- | --- |
|  |  |  |  |  | Start | Stop | Length (bp) | Start | Stop | Anti-condon |
| tRNAPhe | tRNA | F | H |  | 1 | 69 | 69 |  |  | GAA |
| 12S rRNA | rRNA |  | H |  | 70 | 1025 | 956 |  |  |  |
| tRNAVal | tRNA | V | H |  | 1027 | 1098 | 72 |  |  | TAC |
| 16S rRNA | rRNA |  | H |  | 1115 | 2774 | 1660 |  |  |  |
| tRNALeu | tRNA | L2 | H |  | 2775 | 2850 | 76 |  |  | TAA |
| ND1 | Protein-coding |  | H | 324 | 2851 | 3825 | 975 | ATG | TAG |  |
| tRNAIle | tRNA | I | H |  | 3830 | 3901 | 72 |  |  | GAT |
| tRNAGln | tRNA | Q | L |  | 3900 | 3970 | 71 |  |  | TTG |
| tRNAMet | tRNA | M | H |  | 3972 | 4040 | 69 |  |  | CAT |
| ND2 | Protein-coding |  | H | 348 | 4041 | 5087 | 1047 | ATG | T(AA) |  |
| tRNATrp | tRNA | W | H |  | 5086 | 5155 | 70 |  |  | TCA |
| tRNAAla | tRNA | A | L |  | 5157 | 5225 | 69 |  |  | TGC |
| tRNAAsn | tRNA | N | L |  | 5227 | 5299 | 73 |  |  | GTT |
| tRNACys | tRNA | C | L |  | 5331 | 5398 | 68 |  |  | GCA |
| tRNATyr | tRNA | Y | L |  | 5399 | 5469 | 71 |  |  | GTA |
| COX1 | Protein-coding |  | H | 516 | 5471 | 7021 | 1551 | GTG | TAA |  |
| tRNASer | tRNA | S2 | L |  | 7022 | 7092 | 71 |  |  | TGA |
| tRNAAsp | tRNA | D | H |  | 7095 | 7165 | 71 |  |  | GTC |
| COX2 | Protein-coding |  | H | 230 | 7173 | 7863 | 691 | ATG | T(AA) |  |
| tRNALys | tRNA | K | H |  | 7864 | 7939 | 76 |  |  | TTT |
| ATP8 | Protein-coding |  | H | 54 | 7941 | 8105 | 165 | ATG | TAA |  |
| ATP6 | Protein-coding |  | H | 227 | 8099 | 8782 | 684 | ATG | TA(A) |  |
| COX3 | Protein-coding |  | H | 261 | 8782 | 9566 | 785 | ATG | T(AA) |  |
| tRNAGly | tRNA | G | H |  | 9566 | 9636 | 71 |  |  | TCC |
| ND3 | Protein-coding |  | H | 116 | 9637 | 9987 | 351 | ATG | T(AA) |  |
| tRNAArg | tRNA | R | H |  | 9986 | 10054 | 69 |  |  | TCG |
| ND4L | Protein-coding |  | H | 98 | 10055 | 10351 | 297 | ATG | TAA |  |
| ND4 | Protein-coding |  | H | 460 | 10345 | 11725 | 1381 | ATG | TA(A) |  |
| tRNAHis | tRNA | H | H |  | 11726 | 11794 | 69 |  |  | GTG |
| tRNASer | tRNA | S1 | H |  | 11795 | 11863 | 69 |  |  | GCT |
| tRNALeu | tRNA | LI | H |  | 11865 | 11937 | 73 |  |  | TAG |
| ND5 | Protein-coding |  | H | 611 | 11938 | 13773 | 1836 | ATG | TAG |  |
| ND6 | Protein-coding |  | L | 173 | 13770 | 14291 | 522 | ATG | TAA |  |
| tRNAGlu | tRNA | E | L |  | 14292 | 14360 | 69 |  |  | TTC |
| CYTB | Protein-coding |  | H | 380 | 14363 | 15503 | 1141 | ATG | T(AA) |  |
| tRNAThr | tRNA | T | H |  | 15504 | 15576 | 73 |  |  | TGT |
| tRNAPro | tRNA | P | L |  | 15576 | 15645 | 70 |  |  | TGG |
| D-loop | Non-coding |  | H |  | 16052 | 16630 | 579 |  |  |  |
