# Supplementary material for: Complete Mitochondrial Genome of Acheilognathus mengyangensis (Cypriniformes, Cyprinidae, and Acheilognathinae): Characterization and Phylogenetic Analysis
Source: Ecol Evol. 2025 Aug 3;15(8):e71909. doi: 10.1002/ece3.71909 (PMC12318612; doi:10.1002/ece3.71909)
Supplement: Supplementary file 10 — Table S3: Base composition analysis of the A. mengyangensis mitogenome regions. [file ECE3-15-e71909-s008.docx]

**Table S3.** Base composition analysis of the *A. mengyangensis* mitogenome regions.

| Region |  | Base composition (%) | | | | |  |  |
| --- | --- | --- | --- | --- | --- | --- | --- | --- |
|  | Total | T | C | A | G | AT(%) | AT-skew | CG-skew |
| ATP6 | 683 | 30.89 | 27.53 | 27.23 | 14.35 | 58.13 | -0.06 | -0.31 |
| ATP8 | 165 | 24.85 | 25.45 | 38.79 | 10.91 | 63.64 | 0.22 | -0.40 |
| COXI | 1551 | 31.21 | 25.34 | 25.21 | 18.25 | 56.42 | -0.11 | -0.16 |
| COXII | 691 | 28.65 | 25.33 | 28.36 | 17.66 | 57.02 | -0.01 | -0.18 |
| COXIII | 784 | 28.83 | 27.55 | 25.38 | 18.24 | 54.21 | -0.06 | -0.20 |
| CYTB | 1141 | 29.97 | 27.17 | 26.99 | 15.86 | 56.97 | -0.05 | -0.26 |
| ND1 | 975 | 28.62 | 28.51 | 27.08 | 15.79 | 55.69 | -0.03 | -0.29 |
| ND2 | 1045 | 26.12 | 31.20 | 28.90 | 13.78 | 55.02 | 0.05 | -0.39 |
| ND3 | 349 | 29.23 | 29.51 | 26.07 | 15.19 | 55.30 | -0.06 | -0.32 |
| ND4 | 1382 | 28.29 | 28.22 | 28.00 | 15.48 | 56.30 | -0.01 | -0.29 |
| ND4L | 297 | 29.97 | 27.95 | 24.92 | 17.17 | 54.88 | -0.09 | -0.24 |
| ND5 | 1836 | 29.52 | 26.85 | 28.81 | 14.81 | 58.33 | -0.01 | -0.29 |
| ND6 | 522 | 36.97 | 14.37 | 17.24 | 31.42 | 54.21 | -0.36 | 0.37 |
| PCGs | 11421 | 29.52 | 26.90 | 26.98 | 16.61 | 56.49 | -0.04 | -0.24 |
| tRNAs | 1562 | 27.53 | 20.74 | 28.94 | 22.79 | 56.47 | 0.02 | 0.05 |
| D-loop | 1127 | 30.79 | 19.88 | 35.85 | 13.49 | 66.64 | 0.08 | -0.19 |
| 12S rRNA | 958 | 19.00 | 27.14 | 31.11 | 22.76 | 50.10 | 0.24 | -0.09 |
| 16S rRNA | 1680 | 21.31 | 22.68 | 34.70 | 21.31 | 56.01 | 0.24 | -0.03 |
| Complete genome | 16779 | 27.21 | 26.28 | 29.52 | 16.99 | 56.73 | 0.04 | -0.21 |
