# Supplementary material for: Complete Mitochondrial Genome of Acheilognathus mengyangensis (Cypriniformes, Cyprinidae, and Acheilognathinae): Characterization and Phylogenetic Analysis
Source: Ecol Evol. 2025 Aug 3;15(8):e71909. doi: 10.1002/ece3.71909 (PMC12318612; doi:10.1002/ece3.71909)
Supplement: Supplementary file 11 — Table S4: Analysis of RSCU for the PCGs of the mitochondrial genome of A. mengyangensis . Note: “*” denotes a stop codon. [file ECE3-15-e71909-s002.docx]

**Table S4.** Analysis of RSCU for the PCGs of the mitochondrial genome of *A. mengyangensis*.

| AA | Codon | Count | RSCU | AA | Codon | Count | RSCU |
| --- | --- | --- | --- | --- | --- | --- | --- |
| Phe | UUU(F) | 172 | 1.29 | Tyr | UAU(Y) | 116 | 1.09 |
|  | UUC(F) | 94 | 0.71 |  | UAC(Y) | 97 | 0.91 |
| Leu | UUA(L) | 144 | 1.36 |  | UAA(*) | 133 | 1.56 |
|  | UUG(L) | 68 | 0.64 |  | UAG(*) | 85 | 1 |
|  | CUU(L) | 124 | 1.17 | His | CAU(H) | 101 | 0.98 |
|  | CUC(L) | 109 | 1.03 |  | CAC(H) | 106 | 1.02 |
|  | CUA(L) | 128 | 1.21 | Gln | CAA(Q) | 140 | 1.38 |
|  | CUG(L) | 63 | 0.59 |  | CAG(Q) | 63 | 0.62 |
| Ile | AUU(I) | 154 | 1.21 | Asn | AAU(N) | 144 | 1.1 |
|  | AUC(I) | 100 | 0.79 |  | AAC(N) | 117 | 0.9 |
| Met | AUA(M) | 110 | 1.26 | Lys | AAA(K) | 169 | 1.33 |
|  | AUG(M) | 65 | 0.74 |  | AAG(K) | 85 | 0.67 |
| Val | GUU(V) | 68 | 1.34 | Asp | GAU(D) | 70 | 0.99 |
|  | GUC(V) | 38 | 0.75 |  | GAC(D) | 71 | 1.01 |
|  | GUA(V) | 67 | 1.32 | Glu | GAA(E) | 93 | 1.27 |
|  | GUG(V) | 30 | 0.59 |  | GAG(E) | 53 | 0.73 |
| Ser | UCU(S) | 103 | 1.32 | Cys | UGU(C) | 45 | 0.84 |
|  | UCC(S) | 93 | 1.19 |  | UGC(C) | 62 | 1.16 |
|  | UCA(S) | 100 | 1.28 | Trp | UGA(W) | 107 | 1.31 |
|  | UCG(S) | 35 | 0.45 |  | UGG(W) | 56 | 0.69 |
| Pro | CCU(P) | 135 | 1.13 | Arg | CGU(R) | 30 | 0.77 |
|  | CCC(P) | 155 | 1.3 |  | CGC(R) | 43 | 1.11 |
|  | CCA(P) | 126 | 1.06 |  | CGA(R) | 48 | 1.24 |
|  | CCG(P) | 60 | 0.5 |  | CGG(R) | 34 | 0.88 |
| Thr | ACU(T) | 101 | 1.04 | Ser | AGU(S) | 53 | 0.68 |
|  | ACC(T) | 110 | 1.14 |  | AGC(S) | 83 | 1.07 |
|  | ACA(T) | 132 | 1.36 |  | AGA(*) | 68 | 0.8 |
|  | ACG(T) | 44 | 0.45 |  | AGG(*) | 55 | 0.65 |
| Ala | GCU(A) | 54 | 0.77 | Gly | GGU(G) | 52 | 0.81 |
|  | GCC(A) | 122 | 1.74 |  | GGC(G) | 69 | 1.07 |
|  | GCA(A) | 82 | 1.17 |  | GGA(G) | 73 | 1.14 |
|  | GCG(A) | 23 | 0.33 |  | GGG(G) | 63 | 0.98 |

Note: “*” denotes a stop codon.
