# Supplementary material for: Complete Mitochondrial Genome of Acheilognathus mengyangensis (Cypriniformes, Cyprinidae, and Acheilognathinae): Characterization and Phylogenetic Analysis
Source: Ecol Evol. 2025 Aug 3;15(8):e71909. doi: 10.1002/ece3.71909 (PMC12318612; doi:10.1002/ece3.71909)
Supplement: Supplementary file 12 — Table S5: Preference for codon usage in the protein‐encoding genes in the mitochondrial genome of A. mengyangensis . [file ECE3-15-e71909-s009.docx]

**Table S5.** Preference for codon usage in the protein-encoding genes in the mitochondrial genome of *A. mengyangensis*.

|  | CAI | ENC | GC | GC3 |
| --- | --- | --- | --- | --- |
| ATP6 | 0.118 | 48.99 | 0.421 | 0.318 |
| ATP8 | 0.191 | 55.09 | 0.374 | 0.313 |
| COXI | 0.171 | 46.12 | 0.440 | 0.363 |
| COXII | 0.186 | 57.39 | 0.433 | 0.368 |
| COXIII | 0.191 | 45.60 | 0.463 | 0.407 |
| CYTB | 0.169 | 42.36 | 0.434 | 0.408 |
| ND1 | 0.130 | 45.82 | 0.446 | 0.374 |
| ND2 | 0.125 | 41.17 | 0.454 | 0.422 |
| ND3 | 0.126 | 36.93 | 0.453 | 0.445 |
| ND4 | 0.127 | 45.69 | 0.441 | 0.372 |
| ND4L | 0.131 | 48.74 | 0.456 | 0.375 |
| ND5 | 0.158 | 51.06 | 0.418 | 0.403 |
| ND6 | 0.132 | 50.06 | 0.462 | 0.421 |
| PCGs | 0.165 | 57.17 | 0.447 | 0.439 |
